# Knowledge, Attitudes, Practices, and Misconceptions Regarding Vitamins in Blood Metabolism and Anemia Among University Students: A One Center Cross‐Sectional Study in Ghana

**DOI:** 10.1002/hsr2.71706

**Published:** 2025-12-31

**Authors:** David Mawutor Donkor, Gideon Owusu, Victoria Essuon‐Sepah, Obed Asamoah, Isabella Anokye, Alfred Kwadwo Sah, Safianu Apalebilah, Patrick Adu, Joseph Boachie

**Affiliations:** ^1^ Department of Medical Laboratory Sciences University of Cape Coast, College of Health and Allied Sciences Cape Coast Ghana; ^2^ Department of Medical Laboratory Science KAAF University College Accra Region Ghana

**Keywords:** anemia, blood formation, nutrition, vitamin deficiency, vitamins

## Abstract

**Background:**

In Sub‐Saharan Africa, anemia is a significant public health issue, affecting individuals of all ages. While prevention efforts focus on infants, children, and pregnant women, adolescents are overlooked often, leading to ongoing challenges. Despite its prevalence, there is a paucity of research on anemia in adults, particularly university students in Ghana. Studying this demographic can improve understanding and inform public health interventions, addressing the unique needs of university populations and ultimately reducing the burden of anemia.

**Aim:**

The aim of this research was to assess the knowledge, attitudes, and practices of newly admitted university students regarding the importance of vitamins in blood formation, renewal, and function. This study targeted newly admitted university students, as they represent a transitional group from adolescence to young adulthood, often experiencing changes in dietary patterns, living arrangements, and lifestyle that may predispose them to nutritional deficiencies and anemia.

**Methodology:**

This study employed a cross‐sectional design, utilizing both quantitative and qualitative data collection methods. About 300 newly admitted students undergoing routine medical screening at the University Hospital were recruited. Data regarding the knowledge, attitude and practices, as well as misconceptions of participants were obtained using a structured questionnaire, whereas clinical data were obtained from their medical records.

**Results:**

Among the 300 participants, anemia prevalence was 40.0%, with 45.3% demonstrating good knowledge and 50.7% positive attitudes and practices. Knowledge differed significantly by program of study (*p* = 0.03), while misconceptions such as believing that ‘men by default have higher vitamin needs’ were strongly associated with anemia (aOR = 2.29, *p* = 0.022).

**Conclusion:**

While nearly half demonstrated good knowledge and positive attitudes and practices, misconceptions, particularly gender‐based beliefs, significantly increased anemia risk. These findings emphasize the need for targeted, campus‐based nutrition education that not only improves knowledge but also actively corrects misconceptions to reduce anemia burden.

## 1 Introduction

Across the globe, it was estimated that an alarming proportion exceeding 30% of the entire population had encountered anemia, with statistics indicating that more than 80% of the overall burden attributable to this health issue was predominantly concentrated within developing nations Benfo et al., [1]. In numerous countries situated within Sub‐Saharan Africa, anemia was recognized as a critical public health concern, manifesting in varying degrees of severity, ranging from moderate to severe levels, highlighting the gravity of this situation [2]. Anemia in university students is of particular concern as it can impair cognitive function, reduce academic performance, and increase fatigue, thereby affecting educational outcomes.

The adverse health consequences stemming from anemia affected individuals across a wide spectrum of age groups and were traced back to a complex interplay of both nutritional and non‐nutritional determinants [3]. The high rates of nutritional lacks, especially regarding essential micronutrients like iron, vitamin B12, and folate, are key factors in the global prevalence of anemia [4, 5, 6].

Preventive measures aimed at reducing the incidence of anemia predominantly focused on vulnerable populations, specifically infants, young children, and pregnant or lactating women, often neglecting the critical demographic of adolescents [7, 8]. As a result, the ongoing repercussions of anemia within the adolescent population continue to pose substantial challenges [9].

The multifaceted and significant roles played by essential vitamins, including their contributions to blood cell formation and their regulatory functions in metabolism, emphasized the urgent necessity for a comprehensive and thorough investigation into their biological effects and implications Saghiri et al., [10]. Deficiencies in vitamins such as B6, B12, folate, and vitamin C impair hemoglobin synthesis and red blood cell production, making them major contributors to anemia Stabler [11].

Ghana stood out as a country facing numerous formidable issues linked to undernutrition, micronutrients deficiencies, creating a serious public health predicament. On a global scale, Ghana was positioned at a concerning 135th place out of 187 countries that exhibited the highest burden of malnutrition Tandoh et al., [12]. A recent cohort in Cape Coast found that anemia prevalence among university students increased from 20% to over 40% within one academic year, largely due to nutritional deficiencies. This troubling occurrence was linked to a lack of sufficient intake of important nutrients such as vitamin C and folate [13].

Recent studies highlight concerning health issues among young individuals, with anemia rates reaching 72.5% and vitamin A deficiency at 35.6%, signaling a pressing public health challenge Egbi [14]. Additionally, research indicates that many university students lack adequate knowledge of essential vitamins, often self‐prescribing supplements without professional guidance, which raises concerns about their understanding of safe vitamin use [15]. Genç et al. [16]. While some recognize the importance of dietary supplements, a significant number self‐prescribe without professional guidance, exposing a gap in their knowledge of safe vitamin use Sekhri, Kaur [17].

Despite satisfactory awareness of certain vitamins, students often exhibit poor dietary attitudes, such as neglecting sun exposure for vitamin D synthesis. This inconsistency highlights the need for well‐structured educational strategies to enhance understanding of vitamins' roles in blood formation and overall health [18].

There is a notable research gap in assessing the knowledge, attitudes, and practices (KAP) of new university students regarding vitamins. Understanding their KAP can pinpoint areas needing intervention, promoting better health education and preventing deficiencies. This study fills a critical gap by evaluating the knowledge, attitudes, practices, and misconceptions of newly admitted university students on vitamins, with the broader implication of informing targeted interventions to prevent anemia in higher education institutions, and contributing to Sustainable Development Goal 3; ensuring good health and well‐being.

## 2 Materials and Methods

### 2.1 Study Design

The study was conducted from September 2024 to December 2024 during routine medical screenings. This study employed a cross‐sectional design, which exploited both quantitative and qualitative data collection methods to gain a comprehensive understanding of the KAP of newly admitted university students.

### 2.2 Research Study Site

The research was conducted at the University of Cape Coast Hospital, which targeted newly admitted students across various disciplines. University of Cape Coast Hospital is situated at the central region of Ghana, Cape Coast. The University of Cape Coast Hospital is one of the renowned hospitals in the Central Region of Ghana, which provides services like Out‐Patient, In‐patient, Obstetric and Gynecological, Family Medicine, Specialist Services (Dental, Ear and Throat, Eye and Physiotherapy), Diagnostic Services (Laboratory, X‐Ray, ECG, Ultrasound Scan), Pharmaceutical Services, Counseling Services (HIV/AIDS, Family and Psycho‐social), Reproductive, Child‐Health and Family Planning and Mortuary Services.

The University Hospital is also tasked with the annual medical screening of newly admitted university students before their matriculation and commencement of the academic year. Therefore, consented newly admitted undergraduate students undergoing their routine medical screening were targeted and enrolled into the study.

### 2.3 Sample Size

A convenient sampling method was used to ensure representation from different faculties or colleges within the university.

The sample size was estimated using the Slovin's formula *n* $=\frac{N}{\left[1+Ne^{2}\right]}$ [19].

Where “*n*” is the number of samples, “*N*” is the total population, and “e” is error of tolerance (5%). Using the number of newly admitted undergraduate students for 2023/2024 academic year, 9059. The number of samples, *n* = 9059/[1 + 9059 ($0.05^{2}$)] is 383, however, 300 students voluntarily consented.

### 2.4 Selection of Participants

A convenient sampling method was used to select consented prospective participants for this study. This approach was used to ensure diversity in academic backgrounds, which helps improve representativeness despite the non‐random nature of the sampling [20]. All consented newly admitted students who visited the University of Cape Coast Hospital for their medical screening were recruited into the study. In all, 300 newly admitted undergraduate students were enrolled in the current study. The participants had generally gained admission to pursue a wide range of undergraduate programs that were either health‐related or non‐health related.

### 2.5 Inclusion and Exclusion Criteria

Participants included newly admitted undergraduate students at the University of Cape Coast who completed their routine medical screening and voluntarily consented to participate. Postgraduate students, students enrolled in distance learning programs, and others with chronic conditions such sickle cell disease that have been diagnosed and may impact hemoglobin levels were all excluded.

### 2.6 Data Collection

#### 2.6.1 Questionnaire Administration

Closed‐ended structured questionnaires, adopted with slight modifications from previous studies [21, 22, 23], were given to the individual participants. The questionnaire assessed the knowledge, attitude and practices of students on vitamins that are crucial for blood formation, renewal and function, as well as some misconceptions about these vitamins. The questionnaire, adapted from Parmenter & Wardle [24], included 12 knowledge items, 8 attitude items, and 6 practice items [24]. Reliability was confirmed in a pilot test of 30 students (Cronbach's α = 0.78 for knowledge, 0.74 for attitude, 0.81 for practice). The questionnaire was written in a relatively simple and plain English Language which was devoid of technical terminologies or jargons.

### 2.7 Anthropometric and Clinical Data Collection

Participants' anthropometrics including weight (kg) and height (cm), blood pressure, pulse rate as well as laboratory data on anemia and associated risk factors including hemoglobin level, sickling status, proteinuria, haemoglobinuria and hematuria were obtained from the students' medical records.

### 2.8 Ethical Consideration

Before this study was conducted, ethical approval was sought from the Institution Review Board (IRB) of the University of Cape Coast; Ethical Clearance ID (UCCIRB/CHAS/2024/111). Informed consent was obtained from prospective participants before enrolled into the study. Likewise, the participants were at liberty to withdraw from the study at any given time. All procedures undertaken were in conformity with the declaration of Helsinki.

### 2.9 Statistical Analysis

All the data were recorded manually in Microsoft Office Excel. For data processing and management, we used the Statistical Package for Social Sciences (SPSS) version 26 (IBM Inco., USA). Quantitative variables were tested for normality using the Shapiro‐Wilk test and presented as median (interquartile range). Qualitative variables were presented as frequencies and percentages. Also, descriptive statistics, Chi square test, Fisher's Exact test, and binary logistic regression analyzes were used to explore the data. Anemia was defined by hemoglobin levels < 12.5 g/dl and < 11.5 g/dl in males and females respectively, according to established guidelines [25]. Statistical significance was established at *p* < 0.05 at 95% confidence interval.

Knowledge score criteria of “I know,” “I have no idea,” and “I do not know” were used to grade the participants' knowledge regarding vitamins and blood formation. A participant was said to have knowledge of a question if they were able to give a correct answer to the question. The overall knowledge performance of the participants was computed by treating the knowledge responses as a continuous variable. Each correct response to a question was awarded a point, and the total sum of correct responses was found. The 33rd and 67th percentiles were calculated as 5 and 8, respectively. Participants that scored between 0 and 5 were classified as having “low knowledge,” those that scored between 5 and 8 were classified as having “moderate knowledge” and those with a value higher than 8 were classified as having “high knowledge.”

### 2.10 Dependent and Independent Variables

Anemia status, which was classified as present (1) or absent (0) depending on the subjects' hemoglobin levels, was the study's dependent variable. Among the independent variables were common vitamin misunderstandings and knowledge, attitudes, and practices (KAP) about vitamins related to blood formation, renewal, and function. A structured questionnaire was used to assess knowledge, and answers were divided into three categories: I agree, I have no idea, and I disagree. Attitudes and practices were measured based on dietary habits, supplement use, and consultation with healthcare providers. Misconceptions were assessed using Yes/No responses. Additional independent variables included age, sex, program of study, and parental profession.

### 2.11 Measurement Tools and Questionnaire Construction

The questionnaire used in this study was adapted from previous validated research.

### 2.12 Cutoff Points for Knowledge, Attitude, and Practice (KAP) Scores

A composite knowledge score was calculated by assigning 1 point for each correct response and 0 for incorrect or “no idea” responses. The total knowledge score ranged from 0 to 12, with knowledge levels categorized using mean value: Poor (< 5), and Good (≥ 5). Attitude and practice scores were similarly categorized based on responses to Likert‐scale items, where a score of < 4 was Poor attitude, and a score ≥ 4 was Good attitude. These cutoff points align with established methods for knowledge classification in health research [26, 27, 28].

### 2.13 Binary Logistic Regression

Binary logistic regression was used to assess associations between sociodemographic variables and three outcomes: knowledge level, misconceptions about anemia, and anemia prevalence. Sociodemographic variables were included because they are recognized determinants of anemia and may confound these relationships; for example, sex is a biological determinant [29], program of study reflects health literacy differences [30], and parent's profession is a proxy for socioeconomic status affecting diet and healthcare access [31]. History of transfusion and vitamin supplement intake were also included as clinical factors that may independently influence anemia status. Marital status and history of pregnancy (if female) were excluded due to low counts that could have yielded unstable estimates. Adjusted odds ratios (aOR) with 95% confidence intervals (CI) were reported to estimate the strength and direction of associations.

### 2.14 Anemia Classification

Anemia was classified according to World Health Organization (WHO) guidelines, with hemoglobin concentrations < 12 g/dL for women and < 13 g/dL for men considered anemic [32].

## 3 Results

### 3.1 Socio‐Demographics and Clinical Characteristics of the Study Participants

Among the 300 participants, females were slightly more represented (164/300, 54.7%) than males (Table 1). The majority were aged 20–30 years (159/300, 53.0%). Anemia was present in 120/300 (40.0%, 95% CI: 0.54–0.66). Approximately 42% had elevated systolic pressure and 31% had elevated diastolic pressure. Notably, 36/300 (12.0%) had sickling trait.

**Table 1:** Socio‐demographics and clinical characteristics of participants.

Variable	*n* (%)
Age (years)	
Below 20	141 (47.0)
Between 20 and 30	159 (53.0)
Sex	
Male	136 (45.3)
Female	164 (54.7)
Marital status	
Single	300 (100)
Married	0
Program of study	
Health related	71 (23.7)
Non‐health related	229 (76.3)
Parents' profession	
Health related	21 (7.0)
Non‐health related	279 (93.0)
History of transfusion	
Yes	21 (7.0)
No	279 (93.0)
History of pregnancy if female	
Yes	3 (1.0)
No	161 (53.7)
Intake of vitamin supplement recommended by physician	
Yes	96 (32.0)
No	204 (68.0)
*Clinical characteristics*	
BMI	
Underweight	50 (16.7)
Normal	200 (66.7)
Obese	11 (3.7)
Overweight	39 (13.0)
Anemia	
Present	120 (40.0)
Absent	180 (60.0)
Sickling	
Positive	36 (12.0)
Negative	264 (88.0)
Hematuria	
Positive	6 (2.0)
Negative	294 (98.0)
Haemoglobinuria	
Positive	7 (2.3)
Negative	293 (97.7)
SBP (mmHg)	
Normal	173 (57.7)
High	127 (42.3)
SBP (mmHg)	
Normal	207 (69.0)
High	93 (31.0)
Hb (g/dl)	12.1 (11.1–13.5)

*Note:* Anemia classification: males < 12.5 g/dl; females < 11.5 g/dl.

Abbreviations: BMI, body mass index; DBP, diastolic blood pressure; SBP, systolic blood pressure.

### 3.2 Knowledge Assessment of Vitamins

Table 2 shows that most students recognized vitamin C sources (253/300, 84.3%) and adolescent female needs (247/300, 82.3%), whereas fewer identified vitamin B12 in animal foods (138/300, 46.0%) or vitamin B6 as essential for hemoglobin formation (136/300, 45.3%).

**Table 2:** Knowledge of students.

Variable	Frequency (%)
Some vitamins are useful for anemia prevention/treatment	
I agree	245 (81.7)
I have no idea	50 (16.7)
I disagree	5 (1.6)
Vitamin B12 is present in animal food products	
I agree	138 (46.0)
I have no idea	152 (50.7)
I disagree	10 (3.3)
Vitamin B9 cannot be found in plant products	
I agree	44 (13.7)
I have no idea	192 (64.0)
I disagree	64 (21.3)
Citrus and tomatoes are rich sources of vitamin C	
I agree	253 (84.3)
I have no idea	33 (11.0)
I disagree	14 (4.7)
Eating green leafy vegetables does not provide the body with vitamin K_1_	
I agree	46 (15.3)
I have no idea	143 (47.7)
I disagree	111 (37.0)
Shortness of breath and dizziness may be as a result of anemia due to deficiency of some vitamins	
I agree	214 (71.3)
I have no idea	74 (24.7)
I disagree	12 (4.0)
Adolescent females may need vitamin supplementation due to regular menstruation	
I agree	247 (82.3)
I have no idea	45 (15.0)
I disagree	8 (2.7)
Mental retardation in individuals may be as a result of vitamin B12	
I agree	86 (28.7)
I have no idea	193 (64.3)
I disagree	21 (7.0)
People who have gum bleeding disorder may be lacking vitamin C	
I agree	176 (58.7)
I have no idea	88 (29.3)
I disagree	36 (12.0)
Vitamin K helps in the clotting of blood to prevent excessive bleeding	
I agree	168 (56.0)
I have no idea	112 (37.3)
I disagree	20 (6.7)
Vitamin B6 can richly be obtained from fish, liver, and other meat sources	
I agree	161 (53.7)
I have no idea	126 (42.0)
I disagree	12 (4.3)
Vitamin B6 helps in hemoglobin formation	
I agree	136 (45.3)
I have no idea	153 (51.0)
I disagree	11 (3.7)

### 3.3 Knowledge, Attitudes and Practices Categories

As presented in Table 3, 136/300 (45.3%) demonstrated good knowledge, while 164/300 (54.7%) had poor knowledge. Attitudes and practices were nearly evenly split, with 152/300 (50.7%) showing good levels.

**Table 3:** Knowledge and attitudes and practices levels of study participants.

	Category	*n* (%)
Knowledge	Poor	164 (54.7)
	Good	136 (45.3)
Attitudes and practices	Poor	148 (49.3)
	Good	152 (50.7)

### 3.4 Association of Knowledge With Sociodemographic Details

Table 4 indicates that non health‐related program students had significantly better knowledge (112/229, 48.9%) compared with non‐health peers (24/71, 33.8%; χ² = 4.99, *p* = 0.03). No significant associations were observed for age, sex, or parental profession.

**Table 4:** Association of knowledge with sociodemographic details.

Variable	Total, *n* (%)	Poor	Good	χ²	*p* value
Age (years)				2.586	0.13
Below 20	141 (447.0)	84 (51.2)	57 (41.9)		
Between 20 and 30	159 (53.0)	80 (48.8)	79 (58.1)		
Sex				2.929	0.10
Male	136 (45.3)	67 (40.9)	59 (50.7)		
Female	164 (54.7)	97 (59.1)	67 (49.3)		
Program of study				4.990	**0.03**
Health related	71 (23.7)	47 (28.7)	24 (17.6)		
Non‐health related	229 (76.3)	117 (71.3)	112 (82.4)		
Parents' profession				0.453	0.65
Health related	21 (7.0)	10 (6.1)	11 (8.1)		
Non‐health related	279 (93.0)	154 (93.9)	125 (91.9)		
History of transfusion				0.048	> 0.99
Yes	21 (7.0)	11 (6.7)	10 (7.4)		
No	279 (93.0)	153 (93.3)	126 (92.6)		
History of pregnancy if female				2.999^α^	0.25^α^
Yes	3 (1.0)	2 (1.2)	1 (0.7)		
No	161 (53.7)	95 (57.5)	68 (48.5)		
Intake of vitamin supplement recommended by physician				0.380	0.62
Yes	96 (32.0)	50 (30.5)	46 (33.8)		
No	204 (68.0)	114 (69.5)	90 (66.2)		

*Note:* α: Fishers Exact test. Bold values indicate statistically significant.

### 3.5 Sociodemographic Factors Influencing Knowledge of Students About Vitamins

According to Table 5, program of study remained the only independent predictor of good knowledge (aOR 1.82, 95% CI: 1.03–3.21, *p* = 0.04), after adjusting for age, sex, and parental profession.

**Table 5:** Predictors of knowledge in multivariable logistic regression analysis.

	Total, *n* (%)	aOR	95% CI	*p*‐value
Age (years)				
Below 20 (Referent)	141 (447.0)			
Between 20 and 30	159 (53.0)	1.342	0.836–2.155	0.22
Sex				
Male	136 (45.3)			
Female	164 (54.7)	0.477	0.039–5.887	0.56
Program of study				
Health related (Referent)	71 (23.7)			
Non‐health related	229 (76.3)	1.820	1.032–3.208	**0.04**
Parents' profession				
Health related (Referent)	21 (7.0)			
Non‐health related	279 (93.0)	0.710	0.274–1.837	0.48
History of transfusion				
Yes (Referent)	21 (7.0)			
No	279 (93.0)	1.133	0.416–3.085	0.81
History of pregnancy if female				
Yes (Referent)	3 (1.0)			
No	161 (53.7)	1.527	0.121–19.246	0.74
Intake of vitamin supplement recommended by physician				
Yes (Referent)	96 (32.0)			
No	204 (68.0)	0.813	0.489–1.353	0.42

*Note:* Bold values indicate statistically significant.

Abbreviations: aOR, adjusted odds ratio; CI, confidence Interval.

### 3.6 Attitudes and Practices of Participants on Vitamins in Blood Formation

Table 6 shows that risky dietary behaviors were common, with 226/300 (75.3%) reporting meal skipping and 121/300 (40.3%) disliking vegetables. Only 68/300 (22.7%) consulted healthcare professionals for guidance, while 239/300 (79.7%) expressed willingness to learn more about vitamins.

**Table 6:** Attitudes and practices of students.

Variable	Frequency (%)
I consult with healthcare professionals regarding my dietary/vitamin intake requirement	
Yes	68 (22.7)
No	232 (77.3)
I sometimes eat non‐nutritious food substances such as clay (“Shile”) or ice etc.	
Yes	95 (31.7)
No	205 (68.3)
I dislike some vegetables because they don't taste great	
Yes	121 (40.3)
No	179 (59.7)
I do not take vitamin supplements because I am unaware of their benefits	
Yes	87 (29.0)
No	213 (71.0)
I do not take vitamin supplements because they are expensive	
Yes	66 (22.0)
No	234 (78.0)
I sometimes avoid animal sources of protein such as fish, liver, meat, etc. because they are expensive.	
Yes	51 (17.0)
No	249 (83.0)
I sometimes skip breakfast, lunch or supper.	
Yes	226 (75.3)
No	74 (24.7)
I am a strict vegetarian	
Yes	15 (5.0)
No	285 (95.0)
I have no idea whether the cereals (rice, oats) I buy/eat are fortified with vitamins	
Yes	126 (42.0)
No	174 (58.0)
Would you be interested in participating in activities that promote awareness about the importance of vitamins among university students?	
Yes	239 (79.7)
No	61 (20.3)

### 3.7 Association Between Anemia Status and Knowledge and Attitudes and Practices

Table 7 indicates that anemic students were significantly more likely to report good practices (72/120, 60.0%) than non‐anemic peers (80/180, 44.4%; χ² = 6.97, *p* = 0.01). Knowledge levels were not associated with anemia status (*p* = 0.478).

**Table 7:** Association between knowledge levels and attitudes and practices with anemia status.

	Total, *n* (%)	Anemia status		X^2^	*p* value
		Anemic	Non anemic		
Knowledge				0.648	0.45
Poor	164 (54.7)	69 (57.5)	95 (52.8)		
Good	136 (45.3)	51 (42.5)	85 (47.2)		
Attitudes and practices				6.970	**0.01**
Poor	148 (49.3)	48 (40.0)	100 (55.8)		
Good	152 (50.7)	72 (60.0)	80 (44.4)		

*Note:* Bold values indicate statistically significant.

### 3.8 Attitudes and Practices About Anemia

As presented in Table 8, attitudes and practices were not significantly associated with age, sex, program of study, or parental profession (all *p* > 0.05).

**Table 8:** Association between attitudes and practices with sociodemographic details.

Variable	Total, *n* (%)	Poor	Good	X^2^	*p*‐value
Age (years)				0.111	0.82
Below 20	141 (447.0)	71 (48.0)	70 (46.1)		
Between 20 and 30	159 (53.0)	77 (52.0)	82 (53.9)		
Sex				0.044	> 0.9
Male	136 (45.3)	68 (45.9)	68 (44.7)		
Female	164 (54.7)	80 (54.1)	84 (55.3)		
Program of study				1.165	0.34
Health related	71 (23.7)	39 (26.4)	32 (21.1)		
Non‐health related	229 (76.3)	109 (73.6)	120 (78.9)		
Parents' profession				1.141	0.37
Health related	21 (7.0)	8 (5.4)	13 (8.6)		
Non‐health related	279 (93.0)	140 (94.6)	139 (91.4)		
History of transfusion				0.379	0.65
Yes	21 (7.0)	9 (6.1)	12 (7.9)		
No	279 (93.0)	139 (93.9)	140 (92.1)		
History of pregnancy if female				0.336	> 0.99
Yes	3 (1.0)	1 (0.7)	2 (1.3)		
No	161 (53.7)	79 (53.4)	82 (53.9)		
Intake of vitamin supplement recommended by physician				0.692	0.46
Yes	96 (32.0)	44 (29.7)	52 (34.2)		
No	204 (68.0)	104 (70.3)	100 (65.8)		

### 3.9 Misconceptions About Vitamins and Anemia

Misconceptions were widespread (Table 9): 250/300 (83.3%) believed that “the more vitamins, the better,” and 229/300 (76.3%) thought vitamins directly provide energy. Importantly, the belief that ‘men have higher vitamin needs than women' was significantly associated with anemia (aOR 2.29, 95% CI: 1.13–4.66, *p* = 0.02). Other misconceptions, including ‘only vegetarians are at risk of deficiency’, were not significant.

**Table 9:** Association between misconceptions and anemia.

Variable	Total, *n* (%)	Present	Absent	aOR	95% CI	*p*‐value
Vitamin deficiencies affect only people from poor homes						
Yes	46 (15.3)*	21 (45.7)	25 (54.3)	0.837	0.414–1.691	0.62
No	254 (84.7)	99 (39.0)	155 (61.0)			
You have to be skinny to be health						
Yes	29 (9.7)*	12 (41.4)	17 (58.6)	0.714	0.286–1.783	0.47
No	271 (90.3)	108 (39.9)	163 (60.1)			
Only vegetarians are at risk of vitamin deficiency anemia						
Yes	17 (5.7)*	6 (35.3)	11 (664.7)	1.050	0.335–3.293	0.93
No	283 (94.3)	114 (40.3)	169 (59.7)			
Men by default have higher vitamin needs than women						
Yes	58 (19.3)*	17 (29.3)	41 (70.7)	2.294	1.128–4.663	**0.02**
No	242 (80.7)	103 (42.6)	139 (57.4)			
There is one, single food that can ensure good health						
Yes	42 (14.0)*	18 (42.9)	24 (57.1)	0.765	0.364–1.611	0.48
No	258 (86.0)	102 (39.5)	156 (60.5)			
Vitamin deficiencies affect only women						
Yes	13 (4.3)*	6 (46.2)	7 (53.8)	0.626	0.181–2.171	0.46
No	287 (95.7)	114 (39.7)	173 (60.3)			
You cannot get enough vitamins from the food you eat						
Yes	141 (47.0)*	52 (36.9)	89 (63.1)	1.322	0.820–2.131	0.25
No	159 (53.0)	68 (42.8)	91 (57.2)			
Vitamins give you energy						
Yes	229 (76.3)*	91 (39.7)	138 (60.3)	1.170	0.663–2.065	0.59
No	71 (23.7)	29 (40.8)	42 (59.2)			
The more vitamins, the better						
Yes	250 (83.3)*	106 (42.4)	144 (57.6)	0.515	0.256–1.035	0.06
No	50 (16.7)	14 (28.0)	36 (72.0)			
Organic vitamins are better compared to synthetic vitamins						
Yes	233 (77)*	98 (42.1)	135 (57.9)	0.6450	0.354–1.177	0.15
No	67 (22.3)	22 (32.8)	45 (67.2)			

*Note:* Bold values indicate statistically significant.

Abbreviations: aOR, adjusted odds ratio; CI, confidence interval.

* represent participants misconceptions.

### 3.10 Sociodemographic Predictors of Anemia

As shown in Table 10, female sex was independently associated with anemia (aOR 0.11, 95% CI: 0.01–0.26, *p* < 0.01). Age, program of study, parental profession, supplement use, and transfusion history showed no significant associations (all *p* > 0.05).

**Table 10:** Sociodemographic predictors of anemia.

	Total, *n* (%)	Present	Absent	aOR	95% CI	*p*‐value
Age (years)						
Below 20	141 (447.0)	59 (49.2)	82 (45.6)			
Between 20 and 30	159 (53.0)	61 (50.8)	98 (54.4)	0.936	0.552–1.585	0.805
Sex						
Male	136 (45.3)	26 (21.7)	110 (61.1)			
Female	164 (54.7)	94 (78.3)	70 (38.6)	0.112	0.014–0.258	< **0.01**
Program of study						
Health related	71 (23.7)	33 (27.5)	38 (21.1)			
Non‐health related	229 (76.3)	87 (72.5)	142 (78.9)	1.146	0.624–2.105	0.660
Parents' profession						
Health related	21 (7.0)	8 (6.7)	13 (7.2)			
Non‐health related	279 (93.0)	112 (93.3)	167 (92.8)	0.821	0.294–2.295	0.707
Yes	21 (7.0)	9 (7.5)	12 (6.7)			
No	279 (93.0)	111 (92.5)	168 (93.3)	1.736	0.557–5.404	0.341
Intake of vitamin supplement recommended by physician						
Yes	96 (32.0)	46 (38.3)	50 (27.8)			
No	204 (68.0)	74 (61.7)	130 (72.2)	1.349	0.776–2.346	0.289

*Note:* Bold values indicate statistically significant.

## 4 Discussion

In this study of newly admitted university students, we found an anemia prevalence of 40.0%, with 45.3% demonstrating good knowledge of the role of vitamins in blood metabolism and 50.7% showing positive attitudes and practices. Knowledge significantly differed by program of study (*p* = 0.03), while misconceptions such as the belief that “men by default have higher vitamin needs” were significantly associated with anemia (AOR = 2.29, *p* = 0.022). These findings have several implications for nutrition education and public health practice.

### 4.1 Prevalence of Anemia and Knowledge, Attitudes, and Practices (KAP)

The prevalence of anemia (40.0%) among newly admitted students is substantial, indicating a persistent burden in this population. This finding is consistent with a repeated‐measure study among tertiary students in Cape Coast, Ghana, where anemia prevalence more than doubled over the academic year, rising from 20% at entry to nearly 45% by the end [25]. The similarity in magnitude suggests that university students are already vulnerable at admission and may remain at risk throughout their academic years.

Our results also show that although 45.3% of participants demonstrated good knowledge and about half (50.7%) reported positive attitudes and practices, a significant proportion lacked adequate awareness or failed to translate knowledge into healthy behaviors. This mirrors findings from Ghanaian adolescents, where awareness of iron deficiency existed but dietary diversity and intake of iron‐rich foods remained poor Appiah et al., [33]. Such patterns highlight that knowledge alone is insufficient to prevent anemia. The behavioral reinforcement, supportive food environments, and targeted interventions are also necessary.

Interventions should not only aim to improve knowledge but also emphasize behavior change through structural support. Nutrition education could be integrated into secondary and tertiary curricula to establish good practices early, while campus‐based programs could promote access to vitamin‐ and iron‐rich foods. Screening and counseling during student health registrations may also help to identify at‐risk students before anemia worsens.

### 4.2 Role of Misconceptions and Sociodemographic Associations

One of the most important findings of this study is the association between misconceptions and anemia. Students who believed that “men inherently require higher vitamin intake” had more than twice the odds of being anemic. This suggests that even when general knowledge is moderate, entrenched misbeliefs can override correct understanding and lead to harmful practices. Similar observations have been reported in Ghana, where cultural perceptions of anemia often diverge from biomedical explanations and may shape dietary choices Awuah et al., [34].

We also observed that knowledge varied significantly by program of study (*p* = 0.03), with students enrolled in health‐related programs scoring better than their peers. This confirms prior findings that academic exposure influences nutrition literacy (Bawazir, Alrasheedi and Aljehany; Alghamdi et al., [35]). However, the persistence of misconceptions across both groups suggests that formal education alone is insufficient to prevent misinformation.

Targeted myth‐busting interventions are important. Public health campaigns for students should explicitly address common misconceptions, particularly gender‐related beliefs, rather than relying solely on factual instruction. Tailored messaging, informed by formative qualitative research, may be more effective in reshaping attitudes and behaviors. Additionally, interventions could be stratified by program of study, with non‐health‐related students receiving more foundational content.

## 5 Conclusion

This study identified a high prevalence of anemia (40.0%) among newly admitted university students. Although nearly half of participants demonstrated good knowledge (45.3%) and positive attitudes and practices (50.7%), misconceptions, particularly gender‐related beliefs were strongly associated with anemia risk. Knowledge also varied by program of study, with health‐related students performing better, yet misconceptions persisted across groups.

These findings highlight that nutrition interventions in universities must go beyond disseminating factual knowledge. Effective strategies should directly address widespread misconceptions, integrate nutrition education into secondary and tertiary curricula, and ensure supportive food environments that enable healthy practices. Screening at admission and campus‐based counseling may further help reduce anemia burden.

Future research should adopt longitudinal designs to track how student behaviors and nutritional status evolve during university years, and evaluate the impact of tailored interventions, including myth‐busting campaigns and dietary programs on reducing anemia prevalence.

## 6 Limitations

It is vital to acknowledge that the study was conducted at only one location, which raises valid concerns about how broadly the outcomes can be extended to different university demographics that could be diverse in multiple socio‐cultural and demographic factors.

The cross‐sectional approach utilized in this study greatly limits the capacity to definitively determine a causal connection between students' knowledge, attitudes, and practices (KAP) and their related health outcomes, especially concerning the incidence of anemia within the examined group.

Utilizing convenience sampling in this research, while undoubtedly advantageous, could unintentionally lead to some bias, as this method might not fully encompass the rich diversity and variety of the larger student demographic found in the overall educational landscape.

Furthermore, the reliance on self‐reported data for the assessment of knowledge, attitudes, and practices introduces the potential for social desirability bias, wherein participants may feel compelled to overstate their levels of knowledge or to underreport behaviors that are deemed undesirable or socially unacceptable.

## 7 Recommendations

To address knowledge gaps among new university undergraduates about vitamins and their role in blood health, several recommendations are proposed. These include integrating nutrition education into all curricula, with a focus on vitamins and their dietary sources, and creating targeted interventions for high‐risk groups like female students to address specific nutrient needs.

Evidence‐based health campaigns should dispel misconceptions, and universities should improve access to affordable vitamin supplements. Parental involvement in health education is encouraged, alongside regular anemia screening and monitoring. Finally, future research should assess the long‐term impact of these health interventions on students' knowledge and outcomes.

## Author Contributions

**David Mawutor Donkor:** data curation, investigation, methodology, resources, writing – review and editing. **Gideon Owusu:** data curation, investigation, methodology, resources, writing – review and editing. **Victoria Essuon‐Sepah:** data curation, investigation, methodology, resources, writing – review and editing. **Obed Asamoah:** data curation, investigation, methodology, resources, writing – review and editing. **Isabella Anokye:** data curation, investigation, methodology, resources, writing – review and editing. **Alfred Kwadwo Sah:** data curation, investigation, methodology, resources, writing – review and editing. **Safianu Apalebilah:** data curation, investigation, methodology, resources, writing – review and editing. **Patrick Adu:** conceptualization, project administration, supervision, writing – review and editing **Joseph Boachie:** conceptualization, project administration, data curation, investigation, methodology, supervision, writing – review and editing.

## Funding

The authors received no specific funding for this work

## Conflicts of Interest

All authors have read and approved the final version of the article. The corresponding author, Joseph Boachie, had full access to all of the data in this study and takes complete responsibility for the integrity of the data and the accuracy of the data analysis.

## Transparency Statement

The lead author Joseph Boachie affirms that this article is an honest, accurate, and transparent account of the study being reported; that no important aspects of the study have been omitted; and that any discrepancies from the study as planned (and, if relevant, registered) have been explained.

## Data Availability

All data will be made available to interested individuals upon request from the corresponding author.
